# Physical and Recreational Activities, Sedentary Screen Time, Time Spent with Parents and Drug Use in Adolescents

**DOI:** 10.3390/ijerph20021434

**Published:** 2023-01-12

**Authors:** Emanuel Adrian Sârbu, Marius Marici, Simona Bostan, Liviu Gavrila-Ardelean

**Affiliations:** 1 Faculty of Baptist Theology, University of Bucharest, 030018 Bucharest, Romania; 2 Faculty of Sociology and Social Work, University of Bucharest, 010181 Bucharest, Romania; 3 Faculty of Educational Science, Ștefan cel Mare University, 720229 Suceava, Romania; 4 Sociology, Doctoral School of Philosophy, Sociology and Political Sciences, West University of Timisoara, 300223 Timișoara, Romania; 5 Prosthetic Dentistry, Faculty of Dental Medicine, Western University ‘Vasile Goldiș’, 310130 Arad, Romania

**Keywords:** recreational activity, physical activity, sedentary screen time, drug use, time with parents

## Abstract

In a context in which sedentary screen time is on the rise and adolescents are less eager to engage in free-time activities, physical and recreational activities, although too often ignored, have proven to be an antidote for a large array of psychological and behavioral problems in adolescents, including drug use. The present study is a cross-sectional investigation of the association between physical and recreational activities, sedentary screen time, and time spent with parents and the intensity of drug use in adolescents. The participants were part of a representative sample of 2677 adolescents from Bucharest, Romania. The results indicate that vigorous physical and recreational activities, as well as time spent with parents, were negatively associated with an index of drug use (13 drugs), while screen time positively predicted the intensity of drug use. These findings raise the question of the involvement of parents and educational authorities in promoting healthy behaviors and good practices for the prevention of drug use and improving public adolescents’ health.

## 1. Introduction

Romania is a small Eastern European country, which, in the last decade, has been transformed from a transitional market of illicit substances into a market of high-risk drugs, such as heroin [1]. The European Union Report [2] correlated with the European Monitoring Centre for Drugs and Drug Addictions (EMCDDA) Report [3], recognizing Romania as a route for cocaine traffic [2,3]. The drug with the most widespread use by adolescents in Romania remains cannabis [4].

Few studies have been conducted on the situation of drug consumption in Eastern European countries. Kokkevi et al. [5] demonstrated correlations between alcohol and drug dependence in adolescents in Eastern Europe. The study showed ‘particularly strong associations between smoking and going out most evenings’, ‘while cannabis and illegal drugs were strongly correlated with having friends or older siblings who used these substances’ [5] (p. 69). ‘Family structure and societal factors’ [5] (p. 69) (alcohol availability in the country) play an important cultural role in alcoholism and cigarette smoking by adolescents. 

The United Nations Office on Drugs and Crime estimates that, based on the annual report questionnaire data and other official sources, for the Eastern and Southeastern European Regions, in 2020, the annual prevalence of substance use was: 4550 cannabis users, 1720 opioid users, 650 cocaine users (cocaine salt, ‘crack’ cocaine, coca paste, cocaine base, etc.), 570 users of amphetamines and methamphetamine, and 710 ecstasy users [6]. These data are significantly increased compared to the 2017 data according to the Romania Country Drug Report from 2019 [4]. ‘Globally, 80% of adolescents are insufficiently active, and many adolescents engage in 2 h or more daily recreational screen time’ [7] (p. 429). Although parents spend much more time today with their children [8], antisocial behavior, drug use, and other risky behaviors have increased and their prevalence is specific to the transition to adolescence, as shown in cross-sectional and longitudinal research [9,10,11,12,13,14,15,16,17,18,19,20,21,22,23].

We hope that new European programs, such as the EMCDDA Reference Group on drug supply and the European Multidisciplinary Platform Against Criminal Threats (EMPACT), will help the authorities fight against substance abuse through ‘new digital platforms for data collection, recession and drug use and drug consumption rooms’ [24]. We also expect an increase in the prevention of substance use among youth in schools.

## 2. Physical and Recreational Activities in Relation to Drug Use

Studies clearly documenting the association between physical or recreational activities and drug use in adolescents are scarce [25]. Physical activities refer to activities involving ‘bodily movements produced by skeletal muscles that result in energy expenditure’ says a theoretical study [26] (p. 126). The present study addressed vigorous physical activity in reference to sports and aerobics. In a review of the literature, Bardo and Compton [26] found that physical activity, in various forms, reduced marijuana and opioid drug use, and it can serve as a preventive or a treatment measure. The same authors [26] (p. 3) concluded the following: ‘Basic science studies in animal models of drug seeking and drug use risk provide generally strong experimental evidence to conclude that physical activity reduces the acquisition of drug-using behavior and facilitates desistance. Unfortunately, human studies are less clear’. Physical activity was considered a ‘green and environmentally friendly’ [27] (n.p.) treatment method in a study on adults (34.72 ± 9.52 years old). The same study found that ‘Physical activity has a positive effect on reducing drug craving in individuals with SUD’ (n.p.) (Substance Use Disorders). Medium and high physical activity, but not low physical activity, increases internal inhibition (i.e., thoughtfulness and self-control) and has a negative relationship with drug craving (i.e., drug craving, irrational beliefs, and drug cognition) [20] (n.p.). It seems that the effect is dependent on the amount of physical activity. On the one hand, Kwan et al. [28] found that, in a review of the literature on adolescents, in 80% of the studies, sports participation reduced illicit drug use, and in 50% of the studies, marihuana use was negatively associated with sport participation. In 82% of the studies, alcohol use was positively associated with sport participation. On the other hand, it seems that binge drinking and alcohol or even cannabis use have been positively associated with physical activity in the context of competitive sports [20,29]. 

Recreational activities refer to ‘any outdoor activity undertaken for the purpose of exercise, relaxation or pleasure, including practice or instruction in any such activity.’ [30] (n.p.). In their review of the literature, Feldman and Matjasko [31] found that there was a positive relationship between school-based/extracurricular activity participation (i.e., athletics, cheerleading, drama, debate, drilling, music, school newspaper/yearbook, and vocational clubs) and out-of-school activities (i.e., church, community service, and volunteer activities), and a negative relationship with substance use (among other behavioral patterns); the same negative association was also valid for suicidal ideation [32,33]. Extracurricular activities promote developmentally appropriate prosocial behavior of ‘preadolescents and adolescents’ [28,34] (p. 1381) and reduce the likelihood that individuals will engage in risky behavior: ‘students who spend time in extracurricular activities are 49% less likely to use drugs’, ‘marijuana […] cigarette smoking, drinking to get drunk’ [35] (p. 1381). This was contradicted by Veliz, Boyd, and McCabe [36] in their empirical research, which showed that ‘some sporting contexts may be a catalyst to engage in risky behaviors like substance use’ (p. 156). Thus, in the present study, we expect that physical and recreational activity is negatively associated with the frequency of drug use such as smoking marijuana, cocaine, crack, inhalants, and steroids in ‘8th and 10th grade students’ [36] (p. 158). 

## 3. Sedentary Screen Time and Drug Use

Screen time refers to the sedentary or passive use of multimedia devices for entertainment [37]. In recent years, studies have focused on further investigating the relationship between sedentary screen time and other issues, such as drug use or mental health [38]. As a trend, the relationship between sedentary screen time and the intensity of drug use has been poorly documented in the literature, although sedentary screen time has generally been more often correlated with negative psycho-behavioral and even social consequences in adolescents [39,40,41]. 

The existing studies have considered drug use and screen time as comorbidities in nosological disorders, either as behavioral problems or as different behaviors belonging to the same deviant category [42]. Screen time includes the use of various virtual applications, such as chats, video games, video content, internet surfing, and so on. Screen time might be an educational activity or a deviance, as it is often considered, owing to prolonged exposure to it and its consequent negative effects. It is very likely that different legal or illicit drugs are used for stimulation or longer resistance in front of screens, but the opposite could be also true: more drugs could lead to more screen time. It is well known that ‘Performance-enhancing drugs … are commonly used by video gamers.” [43] (n.p.). ‘Those who played under the influence of almost any substance spent more hours per week gaming than the non-users’ [42] (p. 495). In addition, screen time leads to drug use when adolescents do not feel enough pleasure in front of screens [38]. There is ‘a “drug interaction” between self-reinforcing behaviors and addictive substances’ [44] (p. 3980), meaning that the effect of drugs and the pleasure of screen time are reciprocal rewards. 

Although studies on screen time and drug use are scarce, internet use is associated with alcohol use in students [45]. Problematic internet use (i.e., addictive) is associated with drug use (i.e., tobacco, alcohol, cannabis, or other drugs) in adolescents [46]. Thus, we expect that sedentary screen time by adolescents would be positively associated with more drug use. 

## 4. Time with Parents and Child Drug Use

It is a well-known fact that parents and even peers have an important effect on adolescents’ behaviors and their substance use, including that of cigarettes, alcohol, marijuana, and other illegal drugs, such as cocaine and barbiturates [47,48]. Parents are considered to be the moral voices for their children [49], and only a minority of them are deviant and have a bad influence on adolescents’ behavior regarding drug use [50]. Overall, time spent with parents is a protective factor against drug use, as long as it is the framework in which the parent–child relationship develops [51].

On the one hand, in healthy parent–adolescent relationships, they spend enough time with each other, and as a result, the adolescents feel loved and supported. This helps them remain psychologically and physically healthy and achieve optimal development [52]. When parents are present in adolescents’ lives and the parent–adolescent interactions are positive and satisfactory for the adolescents, they are less likely to become involved in substance use. Wills et al. [53] wrote: ‘A positive relationship with parents is posited as a distal factor [value and attitude variable reflecting involvement in conventional goals], presumably because it is associated with more conventional attitudes: [this is] related to substance use in studies of high-school and college students’ (p. 167). 

On the other hand, when one or both parents are absent, the risk of substance use is higher. A study stated that the ‘absence of a father figure is also related to adolescent psychopathology including substance abuse’ [54] (p. 44). Even if the parents are present, distant parent–child relationships or overinvolved and over-controlling parents put children at the same risk of drug use: ‘families of children who misuse drugs were characterized as being those whose fathers were distant and disengaged and whose mothers were too involved’ [55] (p. 204). Even if single parents can definitely have very functional parent–child relationships in all areas, there is a risk that, because of long working hours and insufficient time for family, children are more inclined to engage in deviant behaviors such as drug use: ‘single parents do not have the time to be as supportive and controlling as would parents in intact families’ [56] (p. 321). Thus, time spent with parents in prosocial activities might reduce the risk of drug use.

What is more, in general, adolescents spend less time with their parents than with their same-gender siblings, which could lead to a higher bad influence from their peers, if the latter are deviant [56,57,58]. Spending time together could mean a chance for parents and children to disclose their feelings and thoughts, and parents could better exert their role as advisers. Levine and Singer [59] ‘assessed whom adolescents turn to for help for problems with alcohol or drugs: 84% said they would turn to a friend for help, 66% would turn to a sibling, 41% to their fathers, and 55% to their mothers; female adolescents were more likely to seek help from others’ (p. 19). Spending time together allows parents to monitor and acquire information from and about their children. Thus, they can manage their control and support levels in order to protect adolescents against substance use [60,61,62,63]. Thus, we expect that more time spent with parents on weekends and in after-school activities would play a protective role against drug use.

## 5. Methodology

### 5.1. Present Research

The aim of the present study was to investigate how physical and recreational activities, sedentary screen time, and time spent with parents influence the intensity of drug use in adolescents. The study was a cross-sectional, self-reported, questionnaire-based investigation using a representative sample.

The questionnaire-based study is part of the Youth in Europe—A Drug Prevention Program (or Planet Youth). The model is based on the primary prevention work initiated in Iceland in 1998, which made possible the decrease in substance use amongst Icelandic teenagers to the lowest level compared to all other Western European countries. Starting in 2008, the questionnaire has been applied in Bucharest every other year to a representative sample of 15–16-year-old students [64], based on the research protocol initiated and coordinated by The Icelandic Centre for Social Research and Analysis (ICSRA), Reykjavik University, in partnership with The General Directorate of Social Welfare–Bucharest Municipality. The methodology of the research observes both the ethical considerations of the National Bioethics Committee of Iceland and the national rules and regulations applicable in Romania for the selected age group.

In order to submit the self-completed questionnaire to the selected sample, a protocol was signed by The School Inspectorate of Bucharest Municipality and by each participating school. Whole classes of students were randomly selected, and about 3500 official letters were sent to their parents in order to inform them about the program and the questionnaire and to ask them to allow their minor children to participate in the survey (passive consent). 

To ensure the anonymity and confidentiality of the answers, each participant received their own questionnaire and an empty, self-adhesive envelope to return their answers to the research team. The extensive, omnibus questionnaire included 79 questions on substance use and on a large number of different social factors, aiming to identify the relevant risk and protective factors associated with it.

### 5.2. Participants 

Our sample consisted of 2998 students, aged between 15 and 16 years, who were selected from 84 high schools/colleges in Bucharest. From the entire list of classes in Bucharest school units (mainly in the 10th grade), we randomly selected 108 whole classes of students. They were invited to fill out the questionnaires (which were previously submitted to their teachers along with written instructions for both teachers and students) within two regular weeks of the school year, trying to avoid the periods of winter holidays or the semestrial evaluations/exams. The same core questionnaire (omnibus-type, containing validated scales with variables and indicators referring to areas such as substance use, leisure time activities, wellbeing in school, peer group, family, physical and mental health, etc.) has been used in all of the participating cities. 

The questionnaire has been translated from English into each national language spoken in the participating cities (in our case, Romanian). The translation into Romanian followed the scientific rigor and methodology for translated instruments. The translation tried to use adequate, easy-to-understand language using popular (including colloquial or slang) terms referring to substances (i.e., drugs or pharmaceutical substances) used by teenagers in their daily lives. The teachers received their own questionnaire so they could read the items and help the students in case they needed additional information or clarification during the process. 

At the end of the selected period, 2677 questionnaires were returned. With a small delay, 18 other questionnaires were submitted to the research team for optical scanning and data cleaning, so the final response rate of our sample was 89.9%.

The sample is representative of 10th grade adolescents from the capital of Romania, Bucharest. Bucharest is a city with about 1.79 million people [65].

### 5.3. Instruments 

The instruments selected from the Youth in Europe—A Drug Prevention Program (or Planet Youth)’ and used in the present study are described below:

The Intensity of Drug Use: This item consisted of 13 items, indicating how often adolescents have used various drugs, such as heroin, cocaine, sniffing, etc., in their life. The items were measured on an ordinal scale with seven levels, from ‘never’ (1) to ‘more than 40 times’ (7). Each category in the scale was numerically coded, and we summed up all codes for all 13 items to obtain a total drug score index. The total score index measured the intensity of drug use, indicating the variability in drug use in adolescents (scores ranging from 13 to 91). The total score index was further treated as a continuous variable in the regression analyses used in the present study. There have been statistical studies [66,67,68] that indicate that Likert or ordinal variables with five or more categories can be treated as continuous variables without harming the results. This is often the case in sociological studies based on surveys, such as ours.

Frequency of Time Spent with Parents: This item consisted of two items referring to spending time with parents outside of school hours on working days and spending time with parents during the weekends. The items were measured on a 5-level Likert scale, from 1 (almost never) to 5 (almost always). The score of the frequency of time spent with parents was obtained by summing the scores for each item. The final aggregated variable used in the present study had scores ranging between 2 and 10.

Frequency of Physical Activities Through Sports/Aerobics: This item was measured using four items referring to participating in physical activities: sports and physical training in school, outside of compulsory classes; sports with a sports club/team, exercising and practicing sports outside of school or outside of a club/team; doing physical effort until exhaustion or sweat. The items were measured on an ordinal scale from 0 (almost never) to 6 (almost every day). The final variable was created by summing up all four scores from the four items. The aggregated variable had scores ranging between 0 and 24. 

Time spent in organized recreational/extracurricular activity: This item consisted of a single item asking participants to indicate on a scale from 0 (almost never) to 6 (almost every day) how much time they spend in an organized recreational activity or extracurricular activities. 

Sedentary Screen Time: This item consisted of three items referring to watching video content, spending time on social media, and playing games every day. The items were measured on a scale from 0 (Almost no time) to 8 (6 h or more). The final variable was created by summing up the individual scores from the three items. The aggregated variable had scores between 0 and 24.

### 5.4. Statistical Analyses

In order to analyze the data, we used Jamovi software [69,70]. Jamovi is a free, very intuitive, and open statistical platform that promotes the latest developments in statistics [71]. For the present study, we used descriptive statistics, Spearman correlation, and linear regression analysis. We used linear regression with the ‘Enter’ method, as we have evidence to consider that all these variables might individually explain a part of the variance in ‘the intensity of drug use’. The purpose of the ‘Enter’ method is to show that a newly added variable could predict the outcome of the previous variables.

## 6. Results

### 6.1. Descriptive Statistics

We calculated the descriptive statistics [69] regarding drug use in adolescents in Romania using a representative sample of 2677 participants from Bucharest. As the present database is from a sociological study, it is relevant to provide some descriptive statistics that might help better explain the variables. As the variables in the database were measured on a Likert or a categorical scale, it is most likely to report frequencies, percentages, or cumulative percentages (see Table 1).

### 6.2. Correlation Matrix

In order to determine how the main variables were related, a multiple Spearman correlation analysis in Jamovi [69,70] was performed (see Table 1).

The results (Table 2) indicate that there were significant associations between the intensity of drug use in adolescents and the rest of the variables. Thus, there was a directly proportional relationship between sedentary screen time (r = 0.058, *p* = 0.004) and the intensity of drug use. The results also indicated a negative correlation between time spent by parents with adolescents (r = −0.199, *p* < 0.001), the frequency of physical activity (r = −0.051, *p* = 0.011), or the frequency of recreational activity (r = −0.076, *p* < 0.001) and the intensity of drug use. There was a positive correlation between the frequency of physical activity and the frequency of recreational activity (r = 0.293, *p* < 0.000). The frequency of recreational activity and sedentary screen time were negatively correlated (r = −0.065, *p* < 0.001). There were no significant correlations between the rest of the variables.

### 6.3. Multiple Linear Regression Analysis

To assess the influence of the research variables on the frequency of drug use, a multiple regression analysis was performed. In the first model, the frequency of recreational activity was introduced; in the second model, the frequency of physical activity was added; in the third model, sedentary screen time was added; in the last model, the time spent with parents was added (Table 3). The model fit measures indicate that each model was significant (Table 4) and there were significant differences between all models (Table 5).

The fourth model explained most of the total variance in the frequency of drug use, id est, 5.4% (R^2^_Adj._ = 0.054). 

The results of the analysis of the predictor variable level indicate that the frequency of recreational activity (β = −0.124, *p* < 0.001), the frequency of physical activity (β = −0.085, *p* < 0.001), and time spent with parents significantly predicted the intensity of drug use. The association was negative, meaning that the higher the involvement in recreational or physical activity or the more adolescents spent time with their parents (β = −0.139, *p* < 0.001) the lower the frequency of drug use was. The results also indicate that sedentary screen time was another significant predictor (β = 0.057, *p* < 0.004) of the frequency of drug use, and the association between the two variables was positive. The highest degree of change in the ‘intensity of drug use’ variable for every unit of change in the predictor variable time adolescents spent with their parents was 0.1395 (β). Moreover, for each 1-unit increase in ‘the frequency of recreational activity’, ‘the intensity of drug use’ increased by 0.1245 units. In addition, the ‘intensity of drug use’ changed by 0.0853 units for one unit of ‘the frequency of physical activity’ and by 0.0573 units for the ‘sedentary screen time’ variable. 

Based on the data, the following predictive equation for individual predictions can be inferred. The score for the frequency of drug use = 1.1080 + (−0.0467 × Frequency of recreational activity) + (−0.0290 × Frequency of physical activity) + (0.0172 × Sedentary screen time) + (−0.0613 × Time spent with parents). 

## 7. Discussion

Firstly, recreational and physical activities have generally been positively associated in the literature with well-being and positive and healthy psychological and medical outcomes. A study [72] found that ‘spending 120 min per week in nature is associated with well-being and good health’ (p. 7730). The Leisure Ability Model suggests that the coping skills of drug consumers that increase, such as anhedonia owing to drug use, are replaced by increasing intrinsic positive realities, such as intrinsic motivation, relaxation, or social support connected to recreational activity [73]. 

What is more, time spent in virtual reality playing games, using social media, or watching video content are all predictors of more drug use. The literature has already underlined the negative effects of unhealthy screen time in many studies [39,40,63,74,75] Yet, substance abuse is a correlate of drug activity, although research in the field [42] ‘did not yet reach complete consensus’ (p. 493). The same article indicated that addictions have, to some extent, some common correlates, which explain the positive correlation between drug use and sedentary screen time. 

Finally, time spent with parents was a significant predictor of drug use frequency, but a negative one, meaning that the more time adolescents and parents spent together, the less frequently adolescents consumed drugs. One possible explanation is that parents are moral and prosocial educational vectors and time spent with them is impregnated with desirable social values, which are incompatible with drug use behavior. Time spent with children is a sign of parental love and a huge opportunity to exert control and monitor or set rules for children [51]. In addition, the quantity of time is the basis for quality time, which enriches adolescents’ lives [76].

The results of the present study indicate that all four models and all four variables explained a small percentage of the total variance in the dependent variable, and therefore, we could presume that other variables might better explain the intensity of drug use.

## 8. Conclusions

The aim of the present study was to investigate four antecedents of drug use in adolescents. The results indicate that a higher frequency of recreational and vigorous physical activities and more time spent with parents predicted less drug use, while a higher frequency of sedentary screen time predicted more drug use.

One strong point of this study is that it was performed on a large, representative number of respondents, which allowed for data generalizations. Furthermore, there are few studies that have related drug use and sedentary screen time in adolescents, and thus, this study definitely contributes to the literature. What is more, the study underlines the necessity to measure, in more detail, more psychological realities related to drug use, for example, how drug consumers select free-time activities and how judgements are involved when deciding on one activity over another. The results also suggest the great potential of free-time activities, as directed by specialists [77] who aim at reducing drug use and re-establishing a healthy life.

Future studies should investigate the role of demographic variables regarding drug use and the sex of the user, their socioeconomic status, marital status, employment, and education. In addition, studies should investigate how different degrees of physical activities or recreational activities might influence how adolescents use specific drugs.

## 9. Implications

Modern treatments of drug use are complex, and the approaches are usually multidisciplinary, integrating medical, psychological, and social interventions. Recreational and physical activities are generally associated, in the scientific literature, with physical and mental health benefits, and few studies have advocated for the benefits of physical and recreational preventive measures against drug use. Yet, the present research indicates that vigorous physical activities and recreational activities help and function as protective factors against drug use, in the case of adolescents. In the current context, in which the Romanian school curriculum is overloaded, students work long hours doing their homework together with their parents, and only 28% of children and adolescents (11–15 years) reach a sufficient level of physical activity [78], it is paramount to promote and sustain more consistent physical and recreational activities in the mainstream educational system, in both rural and urban areas. In addition, measures referring to parental involvement by spending time with adolescents in prosocial activities such as sports, while reducing unhealthy screen time and offering love and support, can significantly prevent drug use. 

## Figures and Tables

**Table 1:** Descriptive statistics for the categorical variables in the database: variable name, categories, frequencies, percentages, and cumulative percentages.

Variable	Category	n	%	Cumulative Percentage
Sex	Male	1357	50.4	50.4
	Female	1323	49.1	100
Grade	7th grade	1	0.0	0.0
	8th grade	2	0.1	0.1
	9th grade	58	2.2	2.3
	10th grade	2605	96.7	97.7
Drug use	Never	2479	94.83	94.83
	1–2 times	58	2.22	97.04
	3–5 times	25	0.95	98.00
	6–9 times	13	0.49	98.49
	10–19 times	10	0.39	98.88
	20–39 times	6	0.22	99.10
	40 times or more	24	0.90	100.00
Time spent with parents	Almost never	245	9.16	9.16
	Seldom	405	15.15	24.30
	Sometimes	693	25.93	50.23
	Often	687	25.71	75.94
	Almost always	643	24.06	100.00
Frequency of physical activities through sports/aerobics	Almost never	1394	54.03	54.03
	Once a week	439	17.02	71.05
	Twice a week	458	17.76	88.82
	3 times a week	142	5.49	94.30
	4–6 times a week	89	3.46	97.76
	Almost every day	58	2.24	100.00
Time spent in organized recreational/extracurricular activity	Almost never	1251	48.7	48.7
	Once a week	769	29.9	78.6
	Twice a week	275	10.7	89.3
	3 times a week	118	4.6	93.9
	4–6 times a week	56	2.2	96.1
	Almost every day	101	3.9	100.0
Sedentary screen time	Almost no time	245	9.48	9.48
	½ to 1 h	382	14.79	24.27
	About 1 h	394	15.25	39.52
	About 2 h	516	19.97	59.50
	About 3 h	317	12.27	71.77
	About 4 h	236	9.14	80.90
	About 5 h	131	5.08	85.99
	6 h or more	362	14.01	100.00

Source: Table created with Microsoft Word based on the Jamovi output.

**Table 2:** Spearman correlation matrix.

		**FDU**	**FPA**	**FRA**	**SST**	**TSP**
**FDU**	Spearman’s r_s_	—				
	*p*-value	—				
**FPA**	Spearman’s r_s_	−0.051 *	—			
	*p*-value	0.011	—			
**FRA**	Spearman’s r_s_	−0.076 **	0.293 **	—		
	*p*-value	0.000	0 .000	—		
**SST**	Spearman’s r_s_	0.058 **	−0.004	−0.065 **	—	
	*p*-value	0 .004	0.847	0.001	—	
**TSP**	Spearman’s r_s_	−0.199 **	−0.027	−0.001	−0.007	
	*p*-value	0.000	0.179	0.966	0.711	

Note 1: * *p* < 0.05, ** *p* < 0.01. Note 2: FDU = intensity of drug use, FPA = frequency of physical activity, FRA = frequency of recreational activity, SST = sedentary screen time, TSP = time spent with parents. Note 3: N = 2418. Source: Table created with Microsoft Word based on the Jamovi output.

**Table 3:** Model coefficients resulting from multiple regression analysis.

Predictor	Estimate	SE	t	*p*	Stand. Estimate
MODEL 1					
Intercept	1.0071	0.01731	58.19	<0.001	
Frequency of recreational activity	−0.0610	0.00753	−8.10	<0.001	−0.163
MODEL 2					
Intercept	0.9688	0.02001	48.42	<0.001	
Frequency of recreational activity	−0.0496	0.00809	−6.13	<0.001	−0.1323
Frequency of physical activity	−0.0277	0.00732	−3.78	<0.001	−0.0816
MODEL 3					
Intercept	0.8966	0.03146	28.50	<0.001	
Frequency of recreational activity	−0.0473	0.00812	−5.83	<0.001	−0.1261
Frequency of physical activity	−0.0276	0.00731	−3.78	<0.001	−0.0814
Sedentary screen time	0.0179	0.00603	2.97	0.003	0.0597
MODEL 4					
Intercept	1.1080	0.04324	25.62	<0.001	
Frequency of recreational activity	−0.0467	0.00804	−5.81	<0.001	−0.1245
Frequency of physical activity	−0.0290	0.00724	−4.00	<0.001	−0.0853
Sedentary screen time	0.0172	0.00597	2.88	0.004	0.0573
Time spent with parents	−0.0613	0.00870	−7.05	<0.001	−0.1395

Note: (1) N = 2416, (2) the dependent variable is ‘Intensity of drug use’. Source: Table created with Microsoft Word based on the Jamovi output.

**Table 4:** Model fit measures.

Model	R	R²	Adjusted R²	RMSE
1	0.163	0.026	0.026	0.465
2	0.179	0.032	0.031	0.463
3	0.189	0.036	0.035	0.462
4	0.235	0.055	0.054	0.458

Note: N = 2416; Source: Table created with Microsoft Word based on the Jamovi output.

**Table 5:** Multiple linear regression model comparisons.

Comparison						
Model	Model	ΔR²	F	df1	df2	*p*
1	2	0.00573	14.31	1	2415	<0.001
2	3	0.00352	8.82	1	2414	0.003
3	4	0.01944	49.64	1	2413	<0.001

Note: N = 2416; Source: Table created with Microsoft Word based on the Jamovi output.

## Data Availability

Not applicable.
